# Clinical, procedural and long-term outcome of ischemic VT ablation in patients with previous anterior versus inferior myocardial infarction

**DOI:** 10.1007/s00392-020-01622-z

**Published:** 2020-03-10

**Authors:** Kristina Wasmer, Holger Reinecke, Marius Heitmann, Dirk G. Dechering, Florian Reinke, Philipp S. Lange, Gerrit Frommeyer, Simon Kochhäuser, Patrick Leitz, Lars Eckardt, Julia Köbe

**Affiliations:** 1 Department of Cardiology II - Electrophysiology, University Hospital Muenster, Cardiol, Muenster, Germany; 2 Department of Cardiology I - Coronary and Peripheral Vascular Disease, Heart Failure, University Hospital Muenster, Cardiol, Muenster, Germany

**Keywords:** Ventricular tachycardia, Ischemic cardiomyopathy, Localization of scar, Catheter ablation

## Abstract

**Background:**

Outcome of ischemic VT ablation may differ between patients with previous myocardial infarction (MI) in relation to infarct localization.

**Methods:**

We analyzed procedural data, acute and long-term outcomes of 152 consecutive patients (139 men, mean age 67 ± 9 years) with previous anterior or inferior MI who underwent ischemic VT ablation at our institution between January 2010 and October 2015.

**Results:**

More patients had a history of inferior MI (58%). Mean ejection fraction was significantly lower in anterior MI patients (28 ± 10% vs. 34 ± 10%, *p* < 0.001). NYHA class and presence of comorbidities were not different between the groups. Indication for the procedure was electrical storm in 43% of patients, and frequent implantable cardioverter defibrillator (ICD) therapies in 57%, and did not differ significantly between anterior and inferior MI patients. A mean of 3 ± 2 VT morphologies were inducible, with a trend towards more VT in the anterior MI group (3.1 ± 2.2 vs. 2.6 ± 1.9, *p* = 0.18). Procedural parameters and acute success did not differ between the groups. During a mean follow-up of 3 ± 2 years, more anterior MI patients had undergone a re-ablation (49% vs. 33%, *p* = 0.09, Chi-square test). There was a trend towards more ICD shocks in patients with previous anterior MI (46% vs. 34%). After adjusting for risk factors and ejection fraction, multivariable Cox regression analyses showed no significant difference in mortality (*p* = 0.78) and cardiovascular mortality between infarct localizations (*p* = 0.6).

**Conclusion:**

Clinical characteristics of patients with anterior and inferior MI are similar except for ejection fraction. Patients with inferior MI appear to have better outcome regarding survival, ICD shocks and re-ablation, but this appears to be related to better ejection fraction when compared with anterior MI.

## Introduction

Catheter ablation is an established treatment strategy in patients with structural heart disease to prevent frequent implantable cardioverter defibrillator (ICD) shocks and possibly reduce mortality in these patients [1–5]. Several studies have reported better acute and long-term outcome of catheter ablation in patients with ischemic cardiomyopathy as compared to non-ischemic cardiomyopathy [6, 7]. Substrate localization related to previous anterior or inferior myocardial infarction (MI) may influence parameters related to ventricular tachycardia (VT) ablation including procedural parameters during ablation and acute as well as long-term outcome.

It has been suggested, that inferior scar may be more prone to arrhythmias than anterior scar irrespective of left ventricular ejection fraction (LVEF) [8]. Damage to the inferiorly located parasympathetic nerves in the presence of inferior MI may weaken vagal activity and thereby increase the likelihood of VT. In addition to differences in autonomic innervation, the substrate may differ depending on infarct localization in terms of substrate size and complexity [9, 10] including endocardial and epicardial involvement [11–13].

Based on these pathophysiological considerations we aimed to investigate whether there are differences in patient characteristics, procedural parameters, acute, and long-term outcome between patients with anterior and inferior MI presenting for VT ablation.

## Methods

### Study population

We analyzed the data of consecutive patients with ischemic cardiomyopathy and remote MI who underwent VT ablation at our institution between January 2010 and October 2015. The study was approved by the local research ethics committee (approval number: 2017-309-f-S). On behalf of all authors, the corresponding author states that there is no conflict of interest.

Patients were stratified by whether they had a previous inferior or anterior MI. Infarct localization was determined by patients’ history, results of coronary angiography, inferior or anterior aneurisms on echocardiography and/or magnetic resonance imaging (MRI) and/or Q waves in precordial or inferior leads. Infarct localization was confirmed by scar localization during three-dimensional (3D) mapping. Patients with both, previous anterior and inferior or lateral infarction, or uncertain infarct localization were excluded from analysis.

LVEF was determined by echocardiography in all patients, and by MRI, and/or LV angiography during coronary angiography in some. All patients had stable coronary artery disease, and acute ischemia was excluded in all cases. Clinical parameters including age, sex, time from first and last MI, number of involved coronary vessels, history of coronary artery bypass surgery (CABG), New York Heart Association (NYHA) class, comorbidities, presence and type of defibrillator, time from ICD implantation and ICD-indication as well as medication at the time of presentation were assessed.

### Procedural parameters

We systematically analyzed the indications for ablation, inducibility, number and cycle length (CL) of clinical and induced VT as well as access to the left ventricle (transseptal, retrograde, and epicardial). VT was considered clinical if it was ongoing at the time of the procedure, had the same 12-lead electrocardiogram (ECG) morphology as the documented spontaneous VT (if a 12-lead VT ECG was available), or had the same CL or ≤ 30 ms difference and EGM morphology (if available) matched to that of an ICD-recorded VT. VT was presumed non-clinical, if the previous criteria were not fulfilled. We also analyzed whether activation mapping was used in addition to substrate mapping or substrate mapping only. Indications for VT ablation were defined as either electrical storm, defined as ≥ 3 VT episodes/24 h and incessant VT [14], or frequent ICD interventions [15], defined as ≥ 5 VT episodes in the previous 6 months despite antiarrhythmic drug therapy [16].

### VT ablation procedure

Procedures were done in the fasting state and written informed consent had been obtained prior to the study. Antiarrhythmic medication, with amiodarone and beta-blocker, was not discontinued, while intravenous therapy with ajmaline was discontinued at least six hours prior to the procedure. Conscious sedation, with midazolam, piritramide and disoprivan, was applied based on the investigator’s and the patient’s preference, with continuous invasive arterial blood pressure and oxygen saturation monitoring. In addition, repetitive blood gas analyses including lactate measurements were used for patient monitoring. Implantable defibrillators were deactivated prior to the procedure. Venous access was gained in the left groin with the placement of two sheaths [6 and 7 French (F)]. Two catheters were placed in the right ventricular apex (RVA) and the coronary sinus, respectively, except for patients with cardiac resynchronization devices in whom only a RVA catheter was placed. Venous access to the mapping and ablation catheter (Navistar Thermocool, Biosense Webster Inc., Diamond Bar, CA, USA) was gained in the right femoral groin (8 F). An arterial sheath (5 F) for invasive blood pressure monitoring was placed in the right or left groin. Access to the left ventricle was primarily achieved by transseptal puncture in the majority of cases. If required, additional access was gained retrogradely via the right femoral artery (8 F). Transseptal puncture was performed with a transseptal needle (St. Jude Medical, St. Paul MN, USA) and SL 1 sheath (St. Jude Medical, St. Paul, MN, USA) or steerable sheath (Agilis, St. Jude Medical, St. Paul, MN, USA). Once access to the left heart had been gained, heparin was administered and activated clotting time (ACT) was monitored every 30 min to maintain it above 250 s. 3D mapping (CARTO, Biosense Webster Inc., Diamond Bar, USA) was used in all the cases. In patients with ongoing VT, an activation mapping was performed. In addition to 3D mapping, conventional mapping criteria (entrainment, concealed entrainment, diastolic signals, and fractionated signals) were applied to determine critical sites of the reentrant circuit. In patients who presented with sinus rhythm, VT was induced by programmed ventricular stimulation at different CLs (500, 430, 370, and 330 ms) and for up to three extra-stimuli. If patients tolerated VT (mean invasive blood pressure ≥ 60 mmHg and no lactate during blood gas analysis), VT was targeted by a combination of activation and entrainment mapping. If VT was hemodynamically unstable or not well tolerated (symptoms during light sedation, decreasing blood pressure, increase in lactate level), VT was terminated by pacing or external defibrillator shock delivery on brief deep sedation with disoprivan bolus. A substrate mapping only was performed in patients without inducible VT or patients with hemodynamically unstable VT. Scar was defined as bipolar signal amplitude ≤ 0.5 mV and healthy tissue ≥ 1.5 mV. Values between 0.5 and 1.5 mV were considered as border zones [14].

Radiofrequency energy (RF) was used for ablation with an irrigated tip ablation catheter (Navistar Thermocool, Biosense Webster Inc., Diamond Bar, CA, USA) with a flow rate of 30 ml/min at a maximum of 50 W and maximum temperature set at 45 °C. Ablation targeted all inducible VT. In patients with ongoing VT, RF was delivered over several minutes at the site where VT slowing occurred and extended in a linear fashion to transsect the scar or connect scar to an anatomical barrier, typically the mitral annulus. In patients with hemodynamically unstable VT or several different VT morphologies, ablation was guided by pace mapping (matching QRS morphology, long stimulus QRS interval, local fractionated signals) if VT morphology was available. Scar was defined by a combination of 3D scar mapping and pacing maneuvers to define channels and exits. Substrate ablation was performed by linear ablation extended to anatomic barriers like the mitral annulus, parallel to the scar at sites with fractionated signals and transsecting the scar. Programmed ventricular stimulation was repeated after ablation. It was deferred in patients with hemodynamic instability or in whom VT could not be terminated by the first external defibrillator shock, and after long procedures (> 6 h). Acute ablation outcome was analyzed separately as (1) complete success with no inducible VT, (2) partial success if clinical VT is no longer inducible but non-clinical VT is still inducible, or (3) unsuccessful if the clinical VT was still inducible. ICD were interrogated and reprogrammed after the procedure and pericardial effusion was excluded by echocardiography. Amiodarone was started, continued or discontinued after VT ablation is at the discretion of the treating physician.

### Follow-up

Patients were seen either in the outpatient ICD clinic or by their primary cardiologist every 3 months. Clinical status and ICD interrogation were performed at each visit. In addition, patients and referring physicians were contacted by telephone at the time of manuscript preparation. Patients’ status was obtained including current medication, symptoms, ICD shocks, and re-ablation procedures. Documents of ICD interrogation were collected to assess the VT episodes and ICD therapies. For deceased patients, date of death was documented and cause of death was classified as cardiac (arrhythmic/non-arrhythmic), non-cardiac or unknown.

### Statistical analysis

The cohort was divided into two subgroups according to the location of MI. Categorical variables are presented as absolute numbers (*n*) and percentages (%) of the total numbers for each subgroup; statistical comparisons for these were made by the Chi-squared test. Continuous variables were tested by the ANOVA-*F* test. The impact of the infarct localization on cardiovascular and overall mortality was tested by multivariable Cox regression models adjusted for the number of affected coronary vessels, previous bypass surgery, NYHA class, diabetes, and amiodarone use. Results were displayed as cumulative event curves. The impact of infarct localization on VT recurrence was displayed by Kaplan–Meier model as cumulative event curve. All tests performed were two-sided, and *p* values of < 0.05 were considered statistically significant.

## Results

152 consecutive patients (91% men, mean age 67 ± 9 years, range 36–86 years) with ischemic cardiomyopathy and either anterior or inferior infarction were included in the analysis.

### Patient characteristics

Patient characteristics are summarized in Table 1. There were more patients with inferior than anterior MI (88 (58%) vs. 64 (42%) patients). Ejection fraction was significantly lower in patients with anterior MI (28 ± 10 vs. 34 ± 10, *p* = 0.001), and more patients with inferior compared to anterior MI had undergone (CABG) (47% vs. 30%, *p* = 0.05). Other parameters including sex, age, time from MI, NYHA class, and presence of comorbidities were not significantly different between groups.

**Table 1:** Patient characteristics

	All	Anterior infarction	Inferior infarction	*p* value
No. of patients	152	64	88	
Men (%)	139 (91%)	56 (88%)	83 (94%)	0.14
Age, mean ± SD, years	67 ± 9	67 ± 10	68 ± 9	0.47
CAD				
1-Vessel	21%	28%	15%	0.11
2-Vessel	28%	28%	28%	
3-Vessel	51%	43%	57%	
CABG	59 (39%)	19 (30%)	40 (46%)	**0.049**
Ejection fraction, mean ± SD, %	32 ± 10	28 ± 10	34 ± 10	**0.001**
No. of infarctions				
1	81%	81%	82%	0.994
2	16%	16%	16%	
3	3%	3%	3%	
Time from first infarct, mean ± SD, years	18 ± 8	19 ± 10	17 ± 6	0.482
Time from last infarct, mean ± SD, years	14 ± 10	15 ± 10	13 ± 9	0.351
NYHA class				
I	23%	27%	21%	0.644
II	53%	50%	56%	
III	22%	20%	23%	
IV	2%	3%	1%	
Comorbidities				
Hypertension	83%	78%	86%	0.2
Diabetes	26%	27%	25%	1.0
Atrial fibrillation	43%	45%	42%	0.74
GFR, mean ± SD, ml/min	63 ± 26	67 ± 30	60 ± 23	0.09
ICD at present	133 (88%)	59 (92%)	74 (84%)	0.14
Time since ICD implantation, mean ± SD, years	5.9 ± 4.5	6.2 ± 4.5	5.8 ± 4.5	0.578
Indication for ICD				
Primary prevention	33%	36%	40%	0.72
Types of ICD				
VVI	41%	46%	47%	0.1
DDD	20%	17%	27%	
CRT	27%	37%	26%	
Amiodarone	65%	72%	60%	0.13
Betablocker	92%	92%	92%	1.0

Bold *p*-values are significant (< 0.05)

CAD, coronary artery disease; SD, standard deviation; CABG, coronary artery bypass graft; NYHA, New York Heart Association classification; GFR, glomerular filtration rate; ICD, implantable cardioverter defibrillator; VVI, single chamber ventricular ICD; DDD, dual chamber ICD; CRT, cardiac resynchronization therapy

### Procedural characteristics and acute outcome

Procedural characteristics are summarized in Table 2. The indication for VT ablation was electrical storm in 43% of patients while 57% of patients presented had frequent ICD therapies. This was not different between the two groups. A mean of 2.8 ± 2 VT morphologies were inducible per patient, with a trend towards more inducible VT in previous anterior versus inferior MI. Acute ablation outcome was not significantly different between the groups.

**Table 2:** Procedural parameters and acute outcome

	All	Anterior infarct	Inferior infarct	*p* value
Indication for procedure				
Incessant VT/VT storm	43%	44%	42%	0.83
Frequent ICD therapies	57%	56%	58%	
VT at beginning of procedure	10%	9.4%	10.2%	0.94
Inducible VT	97%	100%	94%	0.08
Clinical VT	65%	45 (70%)	53 (60%)	0.24
Non-clinical VT	72%	48 (75%)	61 (69%)	0.59
No. of inducible VT	2.8 ± 2	3.1 ± 2.2	2.6 ± 1.9	0.18
Clinical VT, CL in ms, mean ± SD	412 ± 87	420 ± 84	407 ± 89	0.46
Access				
Transseptal	95%	91%	98%	0.14
Retrograde	5 (3.3%)	4 (6.3%)	1 (1.1%)	
Epicardial	3 (1.9%)	2 (3.1%)	1 (1.1%)	
Activation map	71%	73%	69%	0.58
Procedure duration, mean ± SD, min	192 ± 82	186 ± 67	196 ± 91	0.443
Time of RF delivery, mean ± SD, min	23 ± 43	26 ± 61	21 ± 23	0.486
Fluoroscopy time, mean ± SD, min	18.8 ± 10	19.4 ± 10.4	18.4 ± 9.8	0.543
No. of ablated VT, mean ± SD	1.5 ± 0.9	1.6 ± 1.0	1.5 ± 0.8	0.59
Stimulation after ablation^a^				
Complete success (no inducible VT)	40%	39%	41%	0.7
Partial success	57%	56%	57%	
Ablation failure (clinical VT still inducible)	3.3%	4.7%	2.3%	

VT, ventricular tachycardia; ICD, implantable cardioverter defibrillator; No., number; CL, cycle length; SD, standard deviation; ms, milliseconds; min, minutes

^a^Stimulation was performed in 127 out of 152 patients (84%)

### Outcome during long-term follow-up

**Fig. 1:**
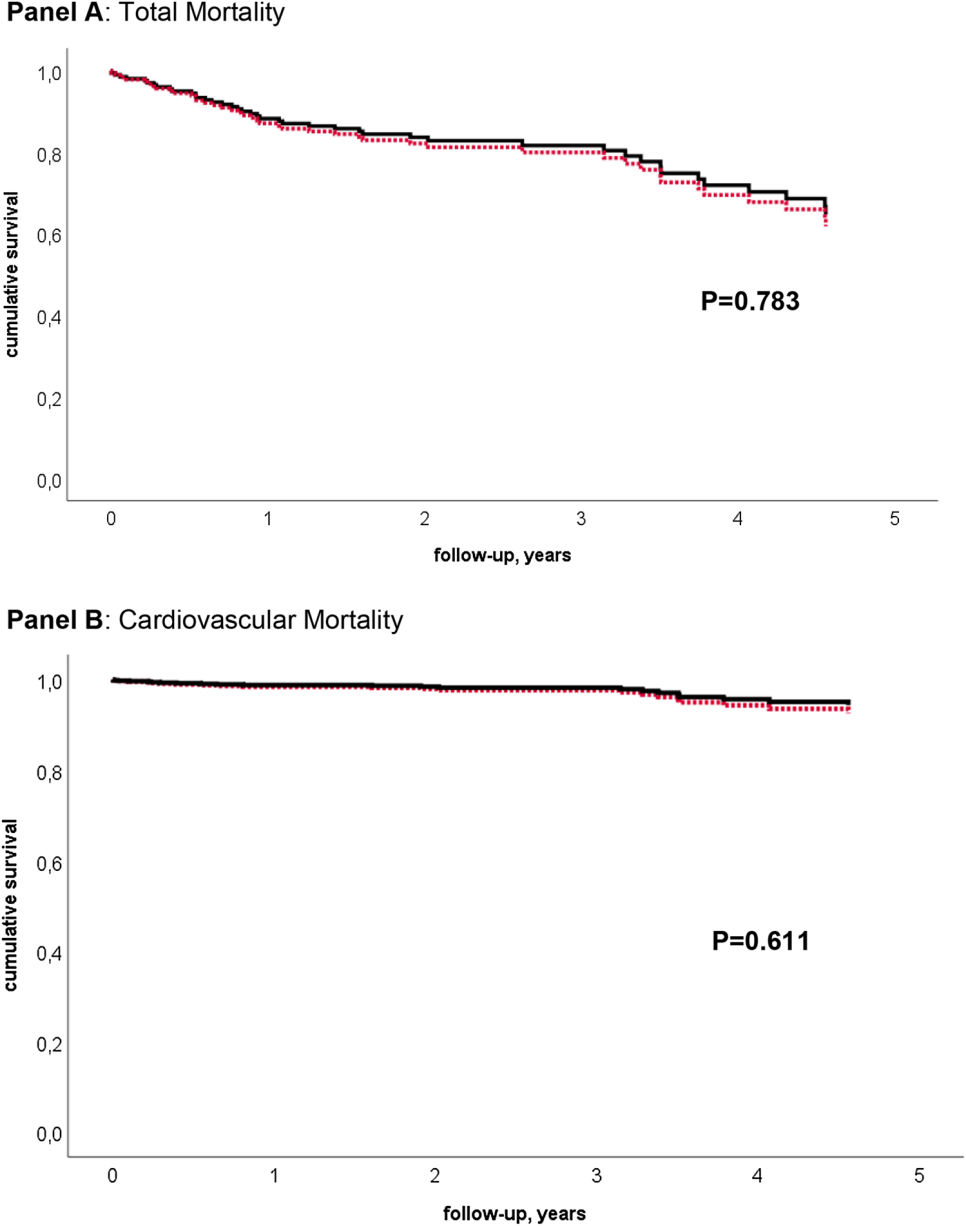
Multivariable Cox regression analyses for total mortality (**a**) and cardiovascular mortality (**b**) during follow-up in patients with inferior myocardial infarction (solid black line) and anterior myocardial infarction (dotted red line). Models were adjusted for number of affected coronary vessels, previous bypass surgery, NYHA class, diabetes, amiodarone use, and ejection fraction

**Fig. 2:**
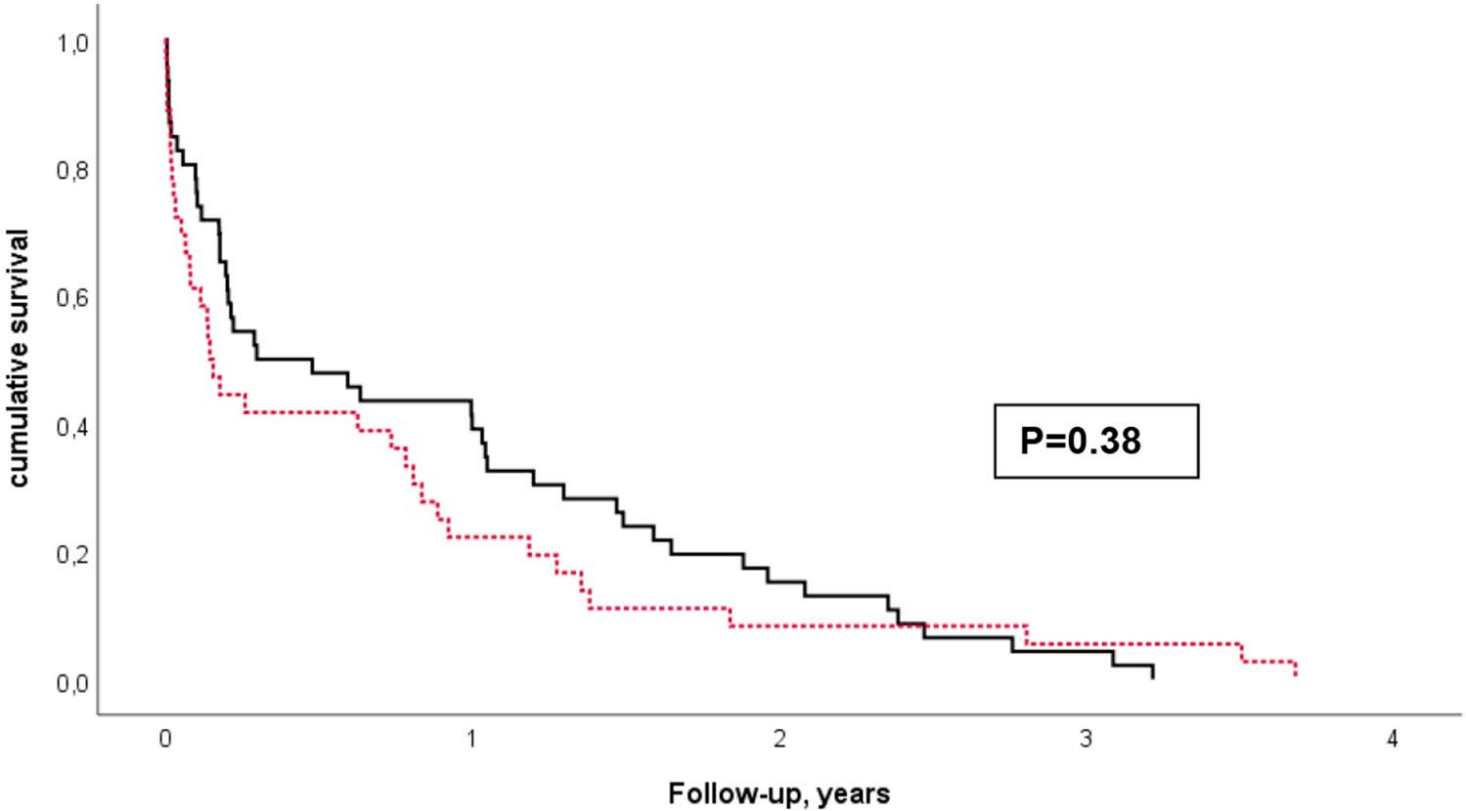
Kaplan–Meier model of VT-free survival during follow-up in patients with inferior myocardial infarction (solid black line) and anterior myocardial infarction (dotted red line)

Patient status was known in 97% of patients after a mean follow-up of 3 ± 2 years (range 0–7 years). More patients with anterior MI compared to inferior MI had died (39% vs. 24%, *p* = 0.062, Chi-square test) with no difference regarding cardiac death between the groups. In addition, cumulative survival between patients with anterior and inferior MI was compared by multivariable Cox regression analysis adjusted for the number of affected coronary vessels, NYHA class, diabetes, previous CABG, and amiodarone use showing a trend for worse outcome in patients with anterior MI regarding total mortality (HR 1.78, 95% CI 0.97–3.28, *p* = 0.065) and cardiovascular mortality (HR 1.90, 95% CI 0.80–4.49, *p* = 0.15). When also adjusted for ejection fraction, there was no difference between infarct localization regarding total mortality (Fig. 1a, HR 1.104, 95% CI 0.55–2.23, *p* = 0.78) and cardiovascular mortality (panel b, HR 0.76, CI 0.26–2.23, *p* = 0.61). More patients with anterior MI compared to inferior MI received ICD shocks during follow-up (46% vs. 34%, *p* = 0.22) and more anterior MI patients had undergone a second VT ablation procedure (49% vs. 33%, *p* = 0.09). Significantly more patients with anterior MI compared to inferior MI patients were using amiodarone (83% vs. 66%, *p* = 0.04). In contrast, there was no significant difference regarding beta-blocker (90% and 93%, *p* = 0.52), ACE-inhibitor and angiotensin II receptor blocker (77% vs. 70%, *p* = 0.42), and aldosterone antagonist use (47% vs. 58%, *p* = 0.2) between anterior and inferior MI patients. VT-free survival is depicted in Fig. 2. There was no significant difference between inferior and anterior MI (*p* = 0.38). We tested whether there was a difference between the patients who presented with electrical storm versus patients with frequent ICD therapies but did not find any differences regarding survival, time of first VT recurrence and ICD shocks. Furthermore, an association between VT inducibility at the end of the procedure and cumulative mortality was assessed by multivariable Cox regression models and found no differences between patients (*p* = 0.64).

## Discussion

The main findings of the present study are

1. a significantly lower LVEF was observed in patients with previous anterior MI compared to those with previous inferior MI with otherwise comparable clinical parameters,
2. comparable procedural parameters and acute ablation success, and
3. a trend towards higher mortality, more ICD shocks and re-ablation during follow-up in patients with previous anterior MI, is no longer observed when adjusting for LVEF.

### Patient characteristics

Previous data suggest that inferior MI may favor the occurrence of VT [8]. In line with this observation, more patients with inferior than anterior MI presented for VT ablation at our center. There may be referral bias and the possibility that anterior MI is associated with worse acute outcome preventing survival to develop late complications like sustained VT. Many studies on ischemic VT ablation do not report localization of the previous infarction. In those that do, though, more patients with inferior than anterior MI were included as observed in our study [7, 18–23].

A lower ejection fraction in patients with anterior infarction has been reported previously [8, 12, 24, 25]. In line with these reports, LVEF in our cohort was significantly lower in patients with anterior MI compared to those with inferior MI. In addition, patients differed with regard to cardiac resynchronization therapy with more biventricular ICD in anterior than inferior MI patients. This supports the notion that patients with previous anterior MI may be sicker than patients with previous inferior MI.

### Mapping and ablation

Different ablation strategies have evolved over the last two decades [26]. Recently, substrate ablation was reported to be superior over ablation of the clinical VT alone [27]. Our mapping and ablation strategy involves a combination of substrate and activation mapping to define the scar and critical parts of the reentrant circuit if VT is ongoing and hemodynamically stable. Our mean RF duration was 24 min, which is somewhat shorter than that in the multicenter study by Stevenson et al. ([23]; 36 min), a study by Sacher et al. ([7]; 33 min), and one by Yamashita et al. ([28]; 32 min). As in other published studies, there is a wide range of RF duration, and studies aiming at complete substrate ablation [27, 28] certainly require more ablation than shorter linear lesions or even focal ablation in a narrow isthmus (mean RF time 32 min, range 20–50 min [28], and 68 ± 21 min [27]).

We use individualized ablation strategies, depending on inducibility, hemodynamic stability, and VT stability. This limits comparison of the ablation approach with other studies beyond acute and long-term success. This is not only a limitation of our analysis but that of VT ablation studies in general. Even apparently standardized strategies like linear ablation or “complete” LAVA ablation are limited by sampling errors and technical limitations (i.e. type and size of mapping catheters, reliable proof of complete linear lesions) [26]. Outcome, on the other hand, is most important for the patient.

### Acute procedural outcome

Endpoints for VT ablation are not clearly defined [29]. Non-inducibility of clinical VT and any VT have both been used to define acute ablation success and are probably the best acute outcome predictors we have. In a meta-analysis, non-inducibility of any VT has been shown to be associated with improved arrhythmia free survival and all-cause mortality [30]. Other data question the predictive value of non-inducibility in post MI patients [29]. In our study, inducibility at the end of the procedure was not clearly associated with an increase in total mortality during follow-up. 40% of patients in our study were non-inducible at the end of VT ablation, while in 57% of patients clinical VT was no longer inducible but non-clinical VT was still inducible. Non-inducibility of any VT ranges between 29 and 93% in the literature [28] with a variable study size (range 15–231 patients). Our results are in the mid-range of these reports. There is certainly a large diversity of patient and substrate characteristics as well as ablation strategies limiting direct comparisons even in prospective randomized studies.

### Long-term outcome

Follow-up was 3 ± 2 years in our study and thereby much longer than in other reports on VT ablation. Follow-up periods typically range between six and 36 months [29]. Recently, Sapp et al. [2] reported outcome data of patients randomized to VT ablation or escalation of medical therapy. During a mean follow-up of 27 ± 17 months, 42% of patients in the catheter ablation group had appropriate ICD shocks and 27% of patients in this group died, which is comparable to our data despite a longer follow-up period in our study. Studies evaluating ablation of late potentials report a VT recurrence rate between 19 and 42% over follow-up periods of 21 and 39 months [29, 32]. In the VISTA trial, 48% of patients with stable VT ablation had VT recurrence during follow-up of 12 months [27]. Of them, 11.9% died during that time. Overall, our outcome data appear to be comparable to those reported in the literature. Recognizing the limitations of a retrospective analysis, our very long-term follow-up data reveal a rather low cardiovascular mortality supporting generous use of VT ablation to improve the quality of life as recommended by current guidelines. In addition, VT that was not induced or targeted during a first procedure may be causing recurrent ICD interventions and trigger a redo procedure rather than recurrence of the same VT. This was described by Tokuda et al. [33] who compared details of repeat procedures to an initial VT ablation. Only 30% of VT were induced at both the initial and the redo procedure. Not only initially unsuccessful or incomplete VT ablation but also changes in the substrate may, therefore, trigger redo procedures irrespective of the ablation strategy used.

### Role of infarct localization

To the best of our knowledge, no study on ischemic VT ablation has yet evaluated the role of infarct localization. As discussed earlier, inferior MI may be more prone to VT based on the patients presenting with VT ablation which is also reflected in a larger proportion of patients with inferior MI in our study. In addition, outcome of patients with anterior MI may be worse compared to inferior MI. This is supported by a recent study that compared the outcome of ischemic mitral regurgitation in anterior versus inferior ST elevation myocardial infarctions (STEMI) [24]. In this study, 30-day, 1-year, and 5-year mortality rates were higher in anterior versus inferior MIs irrespective of the grade of ischemic mitral regurgitation. Differences in quality of scar, e.g. infarct size and density due to early, late or no reperfusion during the initial infarction may also impact the later occurrence of VT and cause differences in outcome. Detailed information regarding reperfusion therapy or enzyme levels during previous infarction was not available for our patient cohort, precluding this analysis. Antz et al. [10] correlated substrate size and ablation success in post MI patients. They reported that 73% of patients with small substrates had a history of inferior infarction that appeared to be better amenable to catheter ablation compared to larger substrates. Recurrence rate, though, did not differ between patients with small, medium and large substrates.

Despite comparable acute ablation outcome in our study, total and cardiovascular mortality were higher in anterior MI patients, and more patients with previous anterior MI presented for a re-ablation procedure. Anterior MI patients also had a significantly lower ejection fraction which may explain worse long-term outcome, irrespective of infarct localization itself. When adjusting for ejection fraction, there was no differences in total mortality and cardiovascular mortality between infarct localizations. The size and quality of scar associated with worse LVEF in anterior versus inferior infarction likely determines patient outcome. Further studies are needed to elucidate the interaction between infarct localization, related quality of scar, ejection fraction, VT incidence and outcome as well as possible underlying mechanisms.

## Limitations

This is a retrospective, non-randomized study with several potential limitations. There is a possibility of referral bias. For example, the patient population consists of a relatively high percentage of VT storm patients which may have influenced acute and long-term outcome. In addition, higher mortality in patients with anterior MI could explain differences in patient numbers presenting for ablation. There is also great diversity in the presentation and course of the procedure. Ablation strategies are, therefore, tailored to the individual patient, limiting comparisons. Nevertheless, our results reveal patient outcome over several years beyond acute results and short follow-up periods.

## Conclusion

Clinical characteristics of patients with previous anterior and inferior MI presenting for VT ablation are similar except for LV function. Patients with inferior MI had better LVEF compared to patients with anterior MI, and they were less often on amiodarone (prior to and after VT ablation). Patients with inferior MI appear to have better outcome regarding survival, shocks during follow-up, and re-ablation. When adjusted for LVEF, though, there were no longer differences in total mortality or cardiovascular mortality between groups, pointing to magnitude of scar and the resulting lower LVEF are most important determinants for patient outcome after VT ablation.

## References

[1] Priori SG, Blomström-Lundqvist C, Mazzanti A (2015). ESC guidelines for the management of patients with ventricular arrhythmias and the prevention of sudden cardiac death: The Task Force for the Management of Patients with Ventricular Arrhythmias and the Prevention of Sudden Cardiac Death of the European Society of Cardiology (ESC). Endorsed by: Association for European Paediatric and Congenital Cardiology (AEPC). Eur Heart J.

[2] Sapp JL, Parkash R, Tang AS (2016). Ventricular tachycardia ablation versus antiarrhythmic-drug escalation. N Engl J Med.

[3] Tung R, Vaseghi M, Frankel DS (2015). Freedom from recurrent ventricular tachycardia after catheter ablation is associated with improved survival in patients with structural heart disease: an International VT Ablation Center Collaborative Group study. Heart Rhythm.

[4] Yokokawa M, Kim HM, Baser K (2015). Predictive value of programmed ventricular stimulation after catheter ablation of post-infarction ventricular tachycardia. J Am Coll Cardiol.

[5] Cronin EM, Bogun FM, Maury P (2019). 2019 HRS/EHRA/APHRS/LAHRS expert consensus statement on catheter ablation of ventricular arrhythmias. Europace.

[6] Dinov B, Fiedler L, Schönbauer R, Bollmann A, Rolf S, Piorkowski C, Hindricks G, Arya A (2014). Outcomes in catheter ablation of ventricular tachycardia in dilated nonischemic cardiomyopathy compared with ischemic cardiomyopathy: results from the Prospective Heart Centre of Leipzig VT (HELP-VT) Study. Circulation.

[7] Sacher F, Tedrow UB, Field ME, Raymond JM, Koplan BA, Epstein LM, Stevenson WG (2008). Ventricular tachycardia ablation: evolution of patients and procedures over 8 years. Circ Arrhythm Electrophysiol.

[8] Pascale P, Schlaepfer J, Oddo M, Schaller MD, Vogt P, Fromer M (2009). Ventricular arrhythmia in coronary artery disease: limits of a risk stratification strategy based on the ejection fraction alone and impact of infarct localization. Europace.

[9] Lacroix D, Warembourg H, Klug D, Decoene C, Kacet S (1999). Intraoperative computerized mapping of ventricular tachycardia: differences between anterior and inferior myocardial infarctions. J Cardiovasc Electrophysiol.

[10] Antz M, Berodt K, Bänsch D, Ernst S, Chun KJ, Satomi K, Schmidt B, Boczor S, Ouyang F, Kuck KH (2008). Catheter-ablation of ventricular tachycardia in patients with coronary artery disease: influence of the endocardial substrate size on clinical outcome. Clin Res Cardiol.

[11] Cesario DA, Vaseghi M, Boyle NG, Fishbein MC, Valderrábano M, Narasimhan C, Wiener I, Shivkumar K (2006). Value of high-density endocardial and epicardial mapping for catheter ablation of hemodynamically unstable ventricular tachycardia. Heart Rhythm.

[12] Yoshiga Y, Mathew S, Wissner E, Tilz R, Fuernkranz A, Metzner A, Rillig A, Konstantinidou M, Igarashi M, Kuck KH, Ouyang F (2012). Correlation between substrate location and ablation strategy in patients with ventricular tachycardia late after myocardial infarction. Heart Rhythm.

[13] Brugada J, Berruezo A, Cuesta A, Osca J, Chueca E, Fosch X, Wayar L, Mont L (2003). Nonsurgical transthoracic epicardial radiofrequency ablation: an alternative in incessant ventricular tachycardia. J Am Coll Cardiol.

[14] Aliot EM, Stevenson WG, Almendral-Garrote JM, European Heart Rhythm Association, European Society of Cardiology, Heart Rhythm Society (2009). EHRA/HRS expert consensus on catheter ablation of ventricular arrhythmias: developed in a partnership with the European Heart Rhythm Association (EHRA), a Registered Branch of the European Society of Cardiology (ESC), and the Heart Rhythm Society (HRS); in collaboration with the American College of Cardiology (ACC) and the American Heart Association (AHA). Europace.

[15] Irvine J, Dorian P, Baker B, O'Brien BJ, Roberts R, Gent M, Newman D, Connolly SJ (2002). Quality of life in the Canadian implantable defibrillator study (CIDS). Am Heart J.

[16] Dev S, Peterson PN, Wang Y, Curtis JP, Varosy PD, Masoudi FA (2014). Prevalence, correlates, and temporal trends in antiarrhythmic drug use at discharge after implantable cardioverter defibrillator placement (from the National Cardiovascular Data Registry [NCDR]). Am J Cardiol.

[17] Callans DJ, Ren JF, Michele J, Marchlinski FE, Dillon SM (1999). Electroanatomic left ventricular mapping in the porcine model of healed anterior myocardial infarction. Correlation with intracardiac echocardiography and pathological analysis. Circulation.

[18] Brunckhorst CB, Stevenson WG, Soejima K, Maisel WH, Delacretaz E, Friedman PL, Ben-Haim SA (2003). Relationship of slow conduction detected by pace-mapping to ventricular tachycardia re-entry circuit sites after infarction. J Am Coll Cardiol.

[19] Fukunaga M, Goya M, Hiroshima K, Hayashi K, Ohe M, Makihara Y, Nagashima M, An Y, Shirai S, Ando K, Yokoi H, Iwabuchi M (2016). Impact of catheter ablation of ventricular tachycardia in patients with prior myocardial infarctions. J Arrhythm.

[20] Mountantonakis SE, Park RE, Frankel DS, Hutchinson MD, Dixit S, Cooper J, Callans D, Marchlinski FE, Gerstenfeld EP (2013). Relationship between voltage map "channels" and the location of critical isthmus sites in patients with post-infarction cardiomyopathy and ventricular tachycardia. J Am Coll Cardiol.

[21] Nakahara S, Tung R, Ramirez RJ, Gima J, Wiener I, Mahajan A, Boyle NG, Shivkumar K (2010). Distribution of late potentials within infarct scars assessed by ultra high-density mapping. Heart Rhythm.

[22] Sarkozy A, Tokuda M, Tedrow UB, Sieria J, Michaud GF, Couper GS, John R, Stevenson WG (2013). Epicardial ablation of ventricular tachycardia in ischemic heart disease. Circ Arrhythm Electrophysiol.

[23] Stevenson WG, Wilber DJ, Natale A, Multicenter Thermocool VT Ablation Trial Investigators (2008). Irrigated radiofrequency catheter ablation guided by electroanatomic mapping for recurrent ventricular tachycardia after myocardial infarction: the multicenter thermocool ventricular tachycardia ablation trial. Circulation.

[24] Mentias A, Raza MQ, Barakat AF, Hill E, Youssef D, Krishnaswamy A, Desai MY, Griffin B, Ellis S, Menon V, Tuzcu EM, Kapadia SR (2016). Outcomes of ischaemic mitral regurgitation in anterior versus inferior ST elevation myocardial infarction. Open Heart.

[25] Kiris I, Kapan S, Narin C, Ozaydın M, Cure MC, Sutcu R, Okutan H (2016). Relationship between site of myocardial infarction, left ventricular function and cytokine levels in patients undergoing coronary artery surgery. Cardiovasc J Afr.

[26] Santangeli P, Marchlinski FE (2016). Substrate mapping for unstable ventricular tachycardia. Heart Rhythm.

[27] Di Biase L, Burkhardt JD, Lakkireddy D (2015). Ablation of stable VTs versus substrate ablation in ischemic cardiomyopathy: the VISTA randomized multicenter trial. J Am Coll Cardiol.

[28] Yamashita S, Cochet H, Sacher F (2016). Impact of new technologies and approaches for post-myocardial infarction ventricular tachycardia ablation during long-term follow-up. Circ Arrhythm Electrophysiol.

[29] Santangeli P, Frankel DS, Marchlinski FE (2014). End points for ablation of scar-related ventricular tachycardia. Circ Arrhythm Electrophysiol.

[30] Ghanbari H, Baser K, Yokokawa M, Stevenson W, Della Bella P, Vergara P, Deneke T, Kuck KH, Kottkamp H, Fei S, Morady F, Bogun F (2014). Noninducibility in postinfarction ventricular tachycardia as an end point for ventricular tachycardia ablation and its effects on outcomes: a meta-analysis. Circ Arrhythm Electrophysiol.

[31] Siontis KC, Kim HM, Stevenson WG (2016). Prognostic impact of the timing of recurrence of infarct-related ventricular tachycardia after catheter ablation. Circ Arrhythm Electrophysiol.

[32] Arenal Á, Hernández J, Calvo D (2013). Safety, long-term results, and predictors of recurrence after complete endocardial ventricular tachycardia substrate ablation in patients with previous myocardial infarction. Am J Cardiol.

[33] Tokuda M, Kojodjojo P, Tung S, Inada K, Matsuo S, Yamane T, Yoshimura M, Tedrow UB, Stevenson WG (2016). Characteristics of clinical and induced ventricular tachycardia throughout multiple ablation procedures. J Cardiovasc Electrophysiol.

